# A Moratorium on Implantable Non-Medical Neurotech Until Effects on the Mind are Properly Understood

**DOI:** 10.1007/s12152-025-09612-6

**Published:** 2025-10-14

**Authors:** Christoph Bublitz, Jennifer A. Chandler, Fruzsina Molnár-Gábor, Marta Sosa Navarro, Philipp Kellmeyer, Surjo R. Soekadar

**Affiliations:** 1 Centre for Law in the Digital Transformation, University of Hamburg, Rothenbaumchaussee 33, 21048 Hamburg, Germany; 2 Center for Health Law, Policy and Ethics, University of Ottawa, Ottawa, Canada; 3 BioQuant Center, Faculty of Law, University of Heidelberg, Heidelberg, Germany; 4 Department of Business and Law, University of Milano-Bicocca, Milan, Italy; 5 Data and Web Science Group, School of Business Informatics and Mathematics, University of Mannheim, Mannheim, Germany; 6 Clinical Neurotechnology Lab, Charité - Universitätsmedizin Berlin, Berlin, Germany

**Keywords:** Technology governance, Mental impact assessment, Brain stimulation, Neuroimaging, Consumer neurotechnology, Digital technology, Human rights based approach, UNESCO, Neuralink

## Abstract

The development of non-medical consumer neurotechnology is gaining momentum. As companies chart the course for future implanted and invasive brain-computer interfaces (BCIs) in non-medical populations, the time has come for concrete steps toward their regulation. We propose three measures: First, a mandatory Mental Impact Assessment that comprehensively screens for adverse mental effects of neurotechnologies under realistic use conditions needs to be developed and implemented. Second, until such an assessment is developed and further ethical concerns are effectively resolved, a moratorium on placing implantable non-medical devices on markets should be established. Third, implantable consumer neurotech for children should be banned. These measures are initial steps in a process seeking to define the necessary requirements for placing these devices on markets. They are grounded in a human rights-based approach to technology regulation that seeks to promote the interests protected by human rights while minimizing the risks posed to them. Neurotechnologies have the potential to profoundly alter cognitive, emotional, and other mental processes, with implications for the rights to mental health and integrity, and possibly for societal dynamics.

## Background

Recent advances in neurotechnologies have sparked interest in developing devices beyond medical purposes. Private investment in consumer devices, mainly from venture capital, has increased significantly, totaling several billion U.S. dollars in the last decade [1]. In May 2025, it was reported that Apple would establish a new input category for controlling its devices — brain signals — alongside touch, typing, and voice [2]. Apple partners with Synchron, a company developing permanently implantable brain-computer interfaces not requiring open-brain surgery. The first use of an iPad through the Stentrode, an electrode grid implanted into blood vessels through a minimally invasive procedure, was demonstrated in August [3, 4]. According to its vision of using “neurotechnology to address the limitations of the human body” [5], the company views this as a “glimpse into the future of human–computer interaction” [4]. It also sees its BCIs as enabling “population-level neural data collection” [6] to train artificial intelligence (AI). In recent months, Neuralink has successfully implanted its N1 device in the first trial participants and is expanding trials to several countries. With the “ultimate goal to create a generalized input/output platform capable of interfacing with every aspect of the human brain” [7], it raised more than 1 billion U.S. dollars in venture capital. Its broader mission is “to unlock human potential”, first of people with unmet medical needs, then of the general population, through increasing the bandwidth of human–computer interaction “by several orders of magnitude”, as its founder Elon Musk explains [8]. The bandwidth increase notably concerns both directions of interaction between brain and device. Brain activity can be transmitted to computers much faster when it is not relayed through slow bodily movements like typing or using a mouse; instead, BCIs read and process brain activity directly. Conversely, human senses are in many ways limited, and directly intervening – or “writing” – in the brain may overcome some of these limitations. While current technologies mainly interact with the motor cortex, deeper brain areas are beginning to be targeted as well. From Neuralink’s perspective, BCIs simply address innate human deficiencies. While one may have doubts about the technical feasibility of these endeavors, the statements unequivocally express the company’s aims and future intended purposes of these devices, and are therefore the basis for ethical and legal assessments.

The BCIs of both companies are in clinical trials with people with disabilities and appear largely functional. Although the stories of patients regaining abilities and acquiring novel ones through their BCIs are raising hopes, it is important to scrutinize and independently verify claims of commercial BCI developers whose underlying research is often neither made public nor peer reviewed. Moreover, at least for Neuralink, these trials are intermediate steps toward the future use of devices in people without disabilities. While at present, there are no compelling applications or interesting use-cases for them, this may change quickly once their functionality expands.

## The Problem

These developments pose serious risks to human rights. Continuing with the bandwidth perspective for a moment, when brains become more closely connected to digital devices, their detrimental effects will likely be exacerbated as well. Key concerns about digital technologies include the weakening of essential cognitive capacities (cognitive deskilling) [9–11], e.g., for attention or sustained focus [12–14], negative impact on well-being and heightened emotional instability [15]. Moreover, the higher bandwidth possible through brain signals amplifies worries about privacy, which most stakeholders have recognized as important, though numerous questions remain [16, 17]. Furthermore, consumer neurotechnologies could leverage brain data to tap into reward-based and other mechanisms for extended and frequent user engagement, as current digital technologies already do. User engagement is integral to many business models, promoting habituation and self-control failures. Many digital technologies tend to promote problematic overuse, which users often regret post factum [18–21]. The empirical picture about many of these effects is still inconclusive, but this is, importantly, not an argument against precautionary risk-based regulation. On the contrary, empirical uncertainties seriously undermine informed use decisions by individuals, and the key objective of our proposal is to improve the epistemic situation.

Moreover, the bandwidth narrative is misleading insofar as it obscures the fact that BCIs not only increase the quantity of inputs and outputs but also open new pathways for signal and stimulus exchange between brain and computer. Currently, mental activity is epistemically largely inaccessible to others. Most information about mental processes is obtained from voluntary expressions, though some is inferred from involuntary reactions such as facial expressions or gestures. Accessing brain activity directly opens a novel source of data about brains and minds, and this source is mostly not under the subject’s control and may contain data about non-conscious processes. This constitutes a qualitatively novel method of gathering data from persons, that calls for heightened transparency and safeguards.

In the other direction, sending stimuli directly into the brain through brain stimulation or other neurointerventions creates a novel input vector for influencing and modifying the brain. As it bypasses the senses and the mechanisms by which incoming sensory stimuli are usually processed, people have considerably less control, if any, over incoming stimuli. The devices thus create a qualitatively new input channel for an “orders of magnitude” larger quantity of stimuli. From this perspective, the attempt to overcome human deficiencies appears to create novel vulnerabilities and susceptibilities.

More generally, the medium and long-term effects of recurrent non-medical brain stimulation on the mind are largely unknown or difficult to ascertain at present [22]. Adverse effects vary between types, parameters, and places of stimulation [23]; current data from non-invasive devices may not generalize to more powerful implantable ones. There is a high prima facie risk of adverse effects that must be mitigated.

The last transformative digital consumer technology, the smartphone, may serve as a cautionary tale. Even after many years, partly due to a lack of high-quality, large-scale, prospective systematic studies, the detrimental effects of specific smartphone applications on cognitive functions are insufficiently understood and optimal modes of use in different contexts such as schools remain controversial (e.g., the recent debate surrounding [24, 25]). Yet this uncertainty has not slowed their roll-out and mass adoption. Furthermore, historical observation teaches us that (digital) technologies often gradually expand into areas of life, work, or society where they were not initially present or needed (“technology creep”), and usually also extend their functions beyond their originally intended use (“function creep”)—digital surveillance technologies being a case in point in recent years [26]. Even though the trajectory of mass adoption seen with the smartphone is not directly transferable to devices involving brain surgery, a laissez-faire approach that awaits consumer adoption is not an adequate way to deal with risks of technologies interfacing directly with the human brain.

## A Human-Rights Based Approach to Neurotechnology

We therefore advocate for a human-rights-based approach to guide the development of invasive consumer neurotechnologies. Technological designs and regulations should promote the interests protected by human rights and minimize risks. Implantable BCIs create or augment risks to privacy, mental health and integrity, which are protected by a trio of human rights. The right to privacy is well-established in human rights law, enshrined, e.g., in article 17 of the International Covenant on Civil and Political Rights, and protects neural data and the information about minds that can be inferred from it. The right to health, article 12(1) of the International Covenant on Economic, Social and Cultural Rights, provides protection against adverse effects on health, including mental health. Finally, the right to mental integrity was first laid down in article 5(1) of the American Convention on Human Rights and more recently in article 3(1) of the European Charter of Fundamental Rights. It aims to protect the mind against undesirable interferences and was adopted in Europe with emerging technologies in mind. While its precise contours still need to be drawn, protecting the mind against barrages of only weakly controllable stimuli likely falls within its scope [27]. In severe cases, even the unconditional right to freedom of thought may be affected. These rights protect different aspects of the human mind – its integrity, health, and privacy – against unwanted interferences. States are obliged to respect, protect, and fulfill these rights, and discharging these obligations requires regulatory efforts.

## Current Regulatory Landscape

While neurotechnologies for medical purposes fall under regulatory frameworks for medical devices, which are based on largely similar values across jurisdictions, the regulation of non-medical devices is heterogeneous and not yet specified in many countries. The European Union (EU) recently regulated non-invasive non-medical brain stimulation devices restrictively, placing them in the highest risk category of the Medical Device Regulation (MDR) [28, 29]. Regulation of implantable devices was deferred. Non-invasive research devices also fall under this category, which has led to fierce criticism from the neuroscience community [30, 31]. This may serve as a warning against stifling research and development. In the U.S., non-medical devices have not yet been subjected to FDA regulation, for now, they fall merely under general laws such as consumer protection laws [22, 32]. Implantable devices might fall under FDA jurisdiction insofar as implantation requires surgery, but no specific regulations have been established. Non-medical devices that only read data from the brain without intervening in it – neuroimaging or one-directional BCIs outside the medical context – are, to our knowledge, not subject to special regulation anywhere and fall only under general product safety and liability laws. Accordingly, implantable non-medical neurotech is nowhere subject to clear, specific, and appropriate regulation. As emerging risks are not sufficiently mitigated, the present legal situation falls short of established human rights obligations.

## Mental Impact Assessment

To overcome epistemic uncertainties about risks, we propose a Mental Impact Assessment that risky neurotechnologies – all implantable non-medical ones – should undergo before being placed on markets. It might be expanded to other digital technologies or limited to some types of neurotechnologies should risks prove acceptable. The Assessment should systematically test for adverse mental effects of neurotechnologies, including more subtle ones on thought and affectivity, under various realistic use scenarios, employing methods suitable for detecting them. Psychological effects are not easily identifiable, and some effects depend on user behavior and interactions with others (as in social media platforms), becoming apparent only at scale. Detecting adverse psychological effects may require complex studies examining subtle changes in subjective experience, mental functioning, and patterns of behavior, with study designs broader than those routinely deployed in clinical trials. They should potentially draw on methods such as phenomenological qualitative research [33], including novel approaches such as microphenomenology. So far, only a few studies have examined the subjective experience of neurotechnology users, particularly in non-clinical contexts (for a review, see [34]). Furthermore, because the frequency and patterns of use of consumer products may differ substantially from medical devices and oversight by physicians is lacking, studies in the clinical context may not generalize to non-medical contexts. At present, the absence of evidence of detrimental effects is not evidence of their absence. Details of the Assessment should be developed through an interdisciplinary effort by academics, clinicians, regulators and industry.

A rigorous methodology to measure the psychological effects of neurotechnology is a sine qua non for any future regulation. In fact, the EU MDR already requires manufacturers of non-invasive brain stimulation devices to “analyse, eliminate, or reduce as far as possible the risks related to: (a) psychological risks; (b) neural and neuro-toxicity risks; [and] (e) long-term side-effect changes in brain functioning.” [29]. This, in our view, requires a comprehensive Mental Impact Assessment. It will also provide the much-needed evidence base for potential subsequent mitigation measures. Without such data, evidence-based and adaptive governance of neurotechnologies appears largely impossible. Patients using implantable neurotech might be an invaluable source of insights in this regard.

The Mental Impact Assessment should be embedded in a regulatory framework for non-medical neurotechnologies that ensures that devices indeed perform as advertised, that risks are acceptable, and that users are fully informed about them and can make genuinely voluntary and fully informed use decisions. It should be conducted before devices are placed on markets. Once released, the genie cannot be put back into the bottle, as subsequent restrictions will face immense hurdles, as the technological lock-in effect in smartphones demonstrates [35]. The alternative is steering technology governance through ex post liability litigation. For instance, a lawsuit is proceeding in Ontario, brought by school boards against social media giants, “alleging platforms have been negligently designed for compulsive use and have rewired the way children think, behave and learn” [36]. However, such legal proceedings take years before a final verdict is reached, require expensive litigation by affected consumers against tech-giants, may shift the burden of proof, and, most importantly, only commence after harm is done, potentially to millions of users. This sets priorities wrongly. We propose testing for such effects before products are rolled out.

A Mental Impact Assessment applies the precautionary principle to real risks posed by neurotechnologies and is the regulatory response to the practice of placing digital technologies on markets without a clear understanding of their mental effects. It protects consumers from carrying the burden of unanticipated adverse effects, much in the same way as with other digital technologies. Pre-market testing must be combined with post-market surveillance for adverse effects detectable only in large-scale and long-term use. Details of regulatory frameworks, such as their being part of medical device regulation or other oversight mechanisms, may vary between countries according to their broader approaches to technology regulation. A legitimate concern is the financing of a Mental Impact Assessment and related research. If costs fall solely on companies, regulation risks fostering monopolization by excluding smaller actors. Public funding with mandatory Open Science publication could therefore be crucial to ensure fairness, transparency, and innovation.

The Mental Impact Assessment should complement privacy assessments, which are already mandatory under certain conditions, e.g., under the EU General Data Protection Regulation. In this context, it should at least be noted that invasive neurotech raises further ethical concerns, e.g., about the use of brain data. Calls for stricter regulations of neurodata have emerged but largely await implementation. It remains to be seen if they are sufficient because they may, e.g., allow tying the access to digital services to the use of neurodata for secondary purposes. And even if they suffice in theory, widespread privacy breaches in the digital sector and the weak enforcement of data protection laws raise concerns about the effectiveness of current approaches.

## Ownership of Mind-Machine Assemblages

Implantable neurotechnologies also raise philosophical questions with concrete ethical and legal implications that need to be resolved. Future devices will operate using adaptive AI methods, particularly machine learning. In so-called closed-loop paradigms, the system may even operate the device without human-in-the-loop user control [37]. When implanted in the body and functionally integrated with the organism, both the hardware and software of the devices merge with the human brain-mind system. According to many accounts, such a hybrid neurotech-AI assemblage thereby becomes part of the body and the person in a strict sense, just like other bodily organs [38, 39]. This creates one of the most intimate conceivable connections between minds, machines, and AI, and marks an intriguing step in human–machine relationships [40]. Human–AI symbiosis is one of Musk’s declared aims for Neuralink. OpenAI is reportedly investing in a startup—Merge Lab—whose name references this aspiration. Whether and under which conditions this boundary should be crossed without medical necessity must be carefully considered and should not be left solely to companies and consumer choice.

Merging minds with machines also raises intriguing questions about the legal status and ownership of implanted devices and the AI that runs them. Should manufacturers retain (intellectual) property rights over devices when they have become part of the body of the person? This raises fears about “app stores” for the brain and monthly subscriptions for “unlocking human potential” [41]. The underlying question is whether companies can have property rights in implanted body parts, and who has the right to control the workings of the implants. The traditional and resounding legal answer that historically emerged from the abolition of slavery is that bodies, or their parts, cannot be owned by others. This principle must be actualized and applied to implantable neurotech and clearly communicated to industry, as it may affect business models.

## Societal Aspects

Beyond individual risks, more powerful neurotechnologies may have detrimental societal effects. Enhancement of cognitive, motivational, or affective features has been debated in the last decade without finding consensus (e.g., [42]). The problem looming large is that their use by some may create peer pressure in specific communities of users, akin to doping in sports [43]. People might be pressured into accepting setbacks to health and integrity for the sake of competitiveness in jobs [44]. In such a competitive environment, individual consumer choice may be effectively limited. This is a situation in which market regulation must secure the safety and efficacy of devices, mitigate side effects, and prevent individuals from unduly endangering themselves for competitive reasons. But where such boundaries should be drawn needs to be determined more precisely.

## A Moratorium for Implantable Consumer Neurotech

Until these and other thorny questions are resolved, details of a Mental Impact Assessment are worked out and empirically tested, the effects of devices on individual minds and society are better understood, and regulatory frameworks with effective institutional structures are in place, we propose a moratorium on placing invasive non-medical neurotechnologies on markets. Moreover, given the development of children's brains, a ban on non-medical neurodevices seems desirable. Unless they can be shown to be largely risk-free, they should be prohibited, as they recently were by the EU (2022, Annex VII, Article 4(1)). The conditions for lifting the moratorium are challenging. In particular, developing working versions of a Mental Impact Assessment may take years of interdisciplinary efforts and should therefore start soon. However, drawing a bright line through a moratorium seems necessary to guide the nascent development of these devices and to limit risks to mental integrity, health, and privacy.

We recognize that moratoria have a mixed track record. Some, such as the prohibition of human germline editing, have gained wide acceptance and guided the development of technologies, whereas others have failed to become practically effective or unduly hampered innovation. At present, the high-stakes risks outweigh the opportunity costs of the moratorium. We fail to see a pressing societal need for invasive non-medical neurotechnologies. Their risks to important human rights, by contrast, are real and not merely based on public misperceptions [45]. Notably, our suggestions should not unduly impair research and development of medical neurotechnologies. Other less burdensome forms of regulation, such as consumer choice models and corporate self-regulation appear insufficient to steer the development of technologies in directions most favorable to individual rights and interests. This is aptly demonstrated by the inability (or unwillingness) of many big tech companies to observe standards such as privacy protection in other digital technologies. There is little reason to assume that this will differ in the present case, where the risks to privacy and mental health are arguably even higher. Since regulation must be pragmatic, the distinction between invasive and non-invasive devices, not free from contestation [46], may serve as a reasonable distinguishing criterion to set the scope of the moratorium. Most invasive neurotech also tends to be considerably more powerful than non-invasive devices, as the latter may suffer from signal attenuation due to the skull and scalp. Finally, moratoria may become ethically problematic when they petrify the status quo without facilitating further productive processes to address concerns [47]. Our moratorium pursues a clear aim, and we invite further discussions of criteria for lifting the moratorium.

Finally, we note the geopolitical dimension of the proposal. As neurotech has become part of the global technological arms race, finding a common global position would facilitate the likelihood of countries adopting such measures. We believe our proposal may be supported by both a large majority of the world population, which tends not to hold transhumanist views, and many governments. The EU passed strict regulations on non-medical non-invasive brain stimulation technologies in 2022, and implantable ones arguably require stricter measures. Last year, China issued Ethics Guidelines for BCI Research. While allowing non-medical “augmentative” BCI research, it calls for “moderation”, “strict regulation and clear benefit” of non-medical devices, minimizing “negative impact on humans”, especially on “human thought” [48]. This seems to require something like a Mental Impact Assessment. The Inter-American Declaration of Principles Regarding Neuroscience, Neurotechnology, and Human Rights by the Organization of American States (2025) contains a principle of “exclusive therapeutic application with respect to enhancement of cognitive abilities” (Principle 6). Rules in the U.S. are more liberal. We understand that the new US administration is reexamining its stance on regulatory and research policies. Our suggestion will not limit the innovation momentum driving the field in recent years because it only pertains to non-medical devices, which play only a peripheral role so far. Their largely unregulated development may lead – over time and at scale – to relevant harms not only to affected individuals but also to the field at large because harms will obstruct the uptake of neurotechnologies in the long run. When it comes to the human mind and brain, responsible innovation must be ensured through sound regulation.

We also note that our proposal finds support in important declarations. In the context of digital technologies, the UN General Assembly has repeatedly acknowledged that the “design, use, deployment and further development of new and emerging technologies (…) may have an impact on the enjoyment of (…) human rights, and that the risks to these rights can and should be avoided and minimized by adapting or adopting adequate regulation or other appropriate mechanisms, (…) and by developing human rights-based auditing mechanisms.” ([49], at 6). Our proposal is consistent with this call, making it specific to mental effects of non-medical neurotechnologies. The OECD Recommendation on Responsible Innovation in Neurotechnology (2019) recommends that member states “prioritise assessing safety in the development and use of neurotechnology”, including “early consideration of potential unforeseen side effects in the research and development of neurotechnologies” (at III 2b). A Mental Impact Assessment would be a formal means to do so.

The 194 member states of UNESCO have just agreed on a Recommendation aiming “to guide neurotechnology into a responsible direction” [50]. It has transpired from the negotiations that the non-medical use of neurotechnologies for enhancement purposes arose as one of the major points of contention that had to remain unresolved. A moratorium would create the necessary breathing space for global deliberations on the conditions for merging human minds with machines. A mandatory comprehensive Mental Impact Assessment and a ban on implantable consumer neurotech for children seem necessary conditions for achieving the aims of the UNESCO document.
